# O056. Migraine as presenting symptom of *SLC20A2*gene mutations

**DOI:** 10.1186/1129-2377-16-S1-A121

**Published:** 2015-09-28

**Authors:** Elisa Rubino, Elisa Giorgio, Innocenzo Rainero, Patrizia Ferrero, Salvatore Gallone, Flora Govone, Lorenzo Pinessi, Laura Orsi, Sergio Duca, Alfredo Brusco

**Affiliations:** Department of Neuroscience “Rita Levi Montalcini”, University of Torino, Turin, Italy; Koelliker Hospital, Turin, Italy; Department of Medical Sciences, University of Torino, Turin, Italy; Department of Neuroscience and Mental Health, A.O.U. Città della Salute e della Scienza, Turin, Italy; Medical Genetics, A.O.U. Città della Salute e della Scienza, Turin, Italy

## Background

Idiopathic basal ganglia calcifications (IBGC), also known as Fahr's disease, are neurological diseases characterized by symmetric calcium deposits in basal ganglia and other brain regions. Clinically, IBGC patients show high phenotypic heterogeneity, both in the clinical manifestations and neuroradiological findings. Recently, *PDGFRB, PDGFB, XPR1* and *SLC20A2* have been identified as causative genes for IBGC[1].

The aim of this study was to report on two Italian patients with idiopathic basal ganglia calcifications associated with novel mutations in the *SLC20A2* gene who both presented with episodic migraine.

## Materials and methods

Two 48-year-old unrelated women presented to the Headache Center, Department of Neuroscience “Rita Levi Montalcini”, University of Torino, with a long-lasting history of headache. The reported symptoms fulfilled ICHD-III beta version criteria for episodic migraine without aura (code 1.1). Computed tomography scans showed in both cases severe calcifications at the bilateral globus pallidus, caudate nuclei, putamen, and dentate nuclei. On the basis of neuroradiological findings, *SLC20A2* gene was sequenced.

## Results

A novel missense mutation Gly63Asp (exon 1) and a frameshift mutation p.Val507Glufs*2 (c.1520_1521delTG, exon 8) in the isoform 1 of the *SLC20A2* gene were identified. *In silico* analysis showed that the first substitution was predicted to have a damaging role. For the frameshift mutation, the genetic variant was found to change an aminoacid and insert a stop codon, likely leading to a degradation of the mutated messenger RNA.

## Discussion and conclusions

The clinical manifestations in patients with IBGC range widely from neurological and psychiatric symptoms to asymptomatic status[2]. We suggest that migraine should be considered when evaluating patients with IBGC and first-degree relatives, in particular in young age, when other neurological symptoms are absent. The identification of new genetic variants further enlarge the spectrum of mutations in *SLC20A2*, helping to better elucidate the worldwide distribution and the different clinical features.

Written informed consent to publication was obtained from the patient(s).
